# Comparison of different salt solutions for density separation of conventional and biodegradable microplastic from solid sample matrices

**DOI:** 10.1007/s11356-022-21474-6

**Published:** 2022-06-22

**Authors:** Berit Schütze, Daniela Thomas, Martin Kraft, Joachim Brunotte, Robert Kreuzig

**Affiliations:** 1 Federal Research Institute for Rural Areas, Forestry and Fisheries, Institute of Agricultural Technology, Thünen Institute, Bundesallee 47, 38116 Brunswick, Germany; 2 Institute of Environmental and Sustainable Chemistry, Technical University of Braunschweig, 38106 Brunswick, Germany

**Keywords:** Microplastics, Sample preparation, Density separation, Digestion

## Abstract

Microplastics are the new emerging pollutants ubiquitously detectable in aquatic and terrestrial ecosystems. Fate and behavior, as well as ecotoxicity, are of increasing environmental concern, particularly in sediments and soils as natural sinks. For a global environmental risk assessment, reliable and easy to apply analytical methods are mandatory to obtain comparable data. This is based on the isolation of microplastics out of the solid sample matrices prior to instrumental detection. Thus, this study provides an easy to apply approach for density separation. The technique emerged from a comparative study using different salt solutions to isolate conventional, and for the first time biodegradable, microplastics from different solid sample matrices, i.e., sand, artificial soil, and compost. Four solutions (water, sodium chloride, sodium hexametaphosphate, and sodium bromide) of different densities were applied followed by oxidizing digestion. Finally, the impact of the procedures on size and surface properties of microplastics was tested. Dependent on the sample matrix, the highest recovery rates of 87.3–100.3% for conventional polymers, and 38.2–78.2% for biodegradable polymers, were determined with sodium bromide. It could be shown that the type of solid sample matrix influences the recovery rates and has to be considered when choosing a sample preparation technique.

**Graphical abstract:**

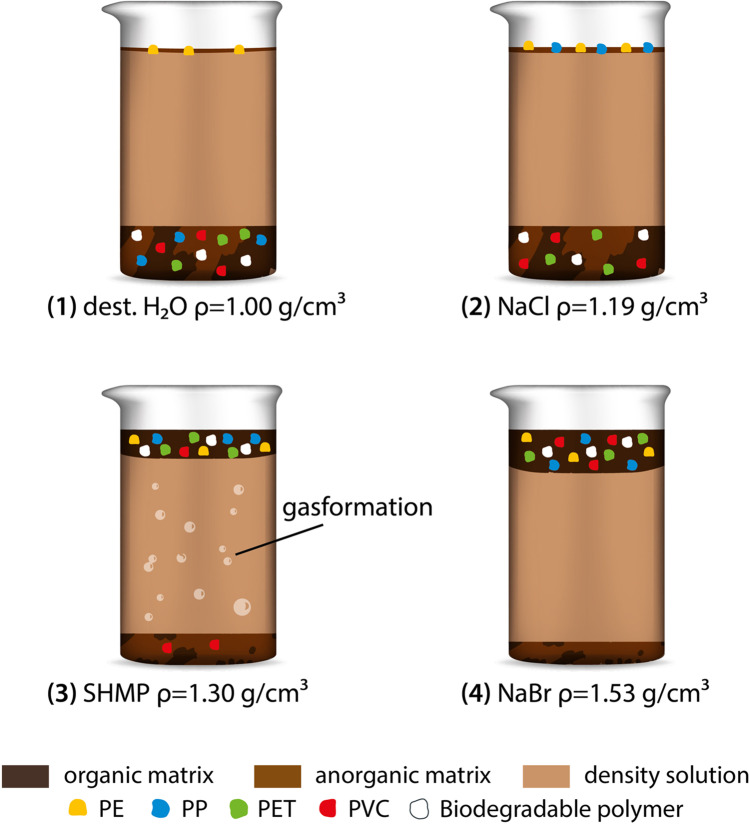

## Introduction

Since the start of the large-scale production of plastic with about 2 Mt (mega tonnes) in the 1950s, production and use have grown rapidly due to benefits like low weight, hygienic use, and easy plasticity (Geyer et al. 2017). In 2019, production of plastics reached a maximum with 368 Mt worldwide (PlasticsEurope 2020). Generally, plastics are released into the environment through either accidental or deliberate pathways of different origins, which results in ubiquitous plastic pollution (Schell et al. 2020). It is estimated that out of the generated 6300 Mt plastic waste up to 2015, nearly 80% were accumulated in the environment (Geyer et al. 2017). Studies confirmed the persistence of plastics in nature (Gewert et al. 2015; Fan et al. 2019) . The main degradation process is assumed to be fragmentation into smaller plastic particles so called “microplastics” (MPs) which are particles with a size between 1 and 1000 μm (Hartmann et al. 2019). However, some definitions contain sizes below 5 mm or 10 mm (Braun et al. 2018).

Additionally, biodegradable plastics have been developed as an alternative to conventional polymers. They have a current share of 0.6% of the global plastic market (PlasticsEurope 2020; Bioplastics 2019). With a share of 43.8%, starch blends are produced the most, followed by polylactid (PLA) with 24.0% and polybutylensuccinate (PBS) with 11.4% of biopolymer production (Haider et al. 2019). Biodegradable alternatives for conventional plastics in mulching for agricultural industry, like MaterBi (MB) by Novamont GmbH (Morra et al. 2016), have been developed. Theoretically, biodegradable plastics will be converted to water and carbon dioxide by biological forces like microorganism metabolism, and can become part of the carbon cycle. However, the degradation potential of plastics strongly depends on the chemical structure (e.g., molecular mass or crystallinity) as well as environmental conditions (e.g., temperature or UV radiation). Number and composition of microorganisms and enzymes present in the corresponding compartment also influence the degradability of plastic (Thakur et al. 2018; Haider et al. 2019). In case of biodegradable polymers, studies suggested that additives will increase the persistency and might make them potentially undegradable under natural conditions (Lambert and Wagner 2017). Recent studies pointed out an even higher risk to the environment of some biodegradable plastics than conventional plastics due to faster fragmentation rates into MPs and incomplete degradation into water and carbon dioxide (Qin et al. 2021; Liao and Chen 2021). Still, formation and effects of biodegradable plastics and MPs to environment and human health are widely unknown.

Sampling strategies, sample preparation methods, and measurement techniques for MP analysis differ strongly within the scientific community (Thomas et al. 2020). In addition, studies rarely identify key factors affecting MP concentrations, such as geographic factors or source dynamics. Currently used techniques to measure MPs lack harmonization and show certain limits, especially for biodegradable MPs, ranging from limited size detection to the limitation of detected polymer types (Büks and Kaupenjohann 2020; O’Kelly et al. 2021). Consequently, previous studies calculated strongly varying concentrations, e.g., of 0.34–690,000 particles/kg soil and < 0.54–67,500 mg plastic/kg in different soil matrices (Zhang et al. 2018; Zhou et al. 2019; Piehl et al. 2018; Fuller and Gautam 2016). Likewise, the effects of MPs on the environment can only be assessed with difficulty. In first studies it was shown that MPs in soil or sediments have an effect on the survival, growth, and reproduction of organisms (de Souza Machado et al. 2018; Boots et al. 2019; Barceló and Picó 2019; Wang et al. 2021; Zhang et al. 2021). It is assumed that MPs accumulate and are transported along trophic levels of the food chain (Huerta Lwanga et al. 2017). MPs are distributed vertically and horizontally within soil. The presence of MPs has potential geochemical- and biophysical-altering effects on soil structure. Their presence might influence soil aggregation, water holding capacity, and soil nutrient cycle (Rillig et al. 2017; Guo et al. 2020). A recent study showed that even biodegradable MPs have a negative effect on soil structure, e.g., by altering soil ecological function and biogeochemical cycling (Zhou et al. 2021).

A harmonized method for the analysis of MP across different solid sample matrices has not yet been established, but is urgently needed to guarantee the comparability of scientific investigations. As there is only a low percentage of MPs in solid samples, MPs need to be isolated from the soil matrix. Since MPs are heterogeneously distributed, mixed samples with the highest possible sample mass will lead to more meaningful results on the content of MPs in solid samples. This in turn leads to increased effort in sample preparation, as organic and inorganic matrices have to be removed for analysis. Especially high organic contents within the sample matrix lead to interfering signals and have to be removed (Thomas et al. 2020). However, many of the previous studies have only processed small sample amounts from 3 to 50 g (Ding et al. 2020; Liu et al. 2019; van den Berg et al. 2020; Scheurer and Bigalke 2018; Corradini et al. 2019). Several techniques can be applied for sample preparation like sieving, visual sorting, extraction, and electrostatic separation, etc. (Quinn et al. 2017; Thomas et al. 2020). The most common techniques are density separation followed by oxidative digestion. Density separation is based on the fact that solid sample matrices have significantly higher specific densities, e.g., bottom sediments (2.65 g/cm^3^), than most plastics (0.05–1.70 g/cm^3^) (Chubarenko et al. 2016). However, organic matter is not separated from plastics with density separation as it shows similar densities of about 1.0–1.6 g/cm^3^ (Bläsing and Amelung 2018; Cerli et al. 2012). Thus, oxidative digestion can be applied. Effects of sample matrices on efficiency of the separation method need to be assessed. Moreover, there is no valid method available for measuring concentrations of biodegradable MPs in solid matrices.

In this study, we aim to generate an easy to apply sample preparation method to detect conventional and biodegradable MPs in solid sample matrices. Therefore, a minimum standard has to be found, which enables a worldwide application to collect comparable data on MPs pollution worldwide. We compared the applicability of different solutions with increasing density for density separation as well as oxidative digestion under various conditions. The solutions used for density separation were water (H_2_O), sodium chloride (NaCl), sodium bromide (NaBr), and sodium hexametaphosphate (SHMP). These are considered to be harmless to humans and the environment according to the Globally Harmonized System of Classification, Labelling and Packaging of Chemicals (GHS). This is considered to be particularly important because the users have different chemical backgrounds. NaCl and NaBr solutions provided high recovery rates of MPs in other studies and are relatively cheap (Scheurer and Bigalke 2018; Liu et al. 2018; Thomas et al. 2020) while SHMP is applied as a dispersing agent for soils and sediments (Scheurer and Bigalke 2018; Vermaire et al. 2017). Recovery experiments were conducted by spiking solid sample matrices with different organic contents with conventional and biodegradable MPs in two separate sample set ups. In addition, the effect of sample preparation on MPs was tested. The effect should be as low as possible, especially when performing further identification tests of MPs.

## Methods

**Fig. 1:**
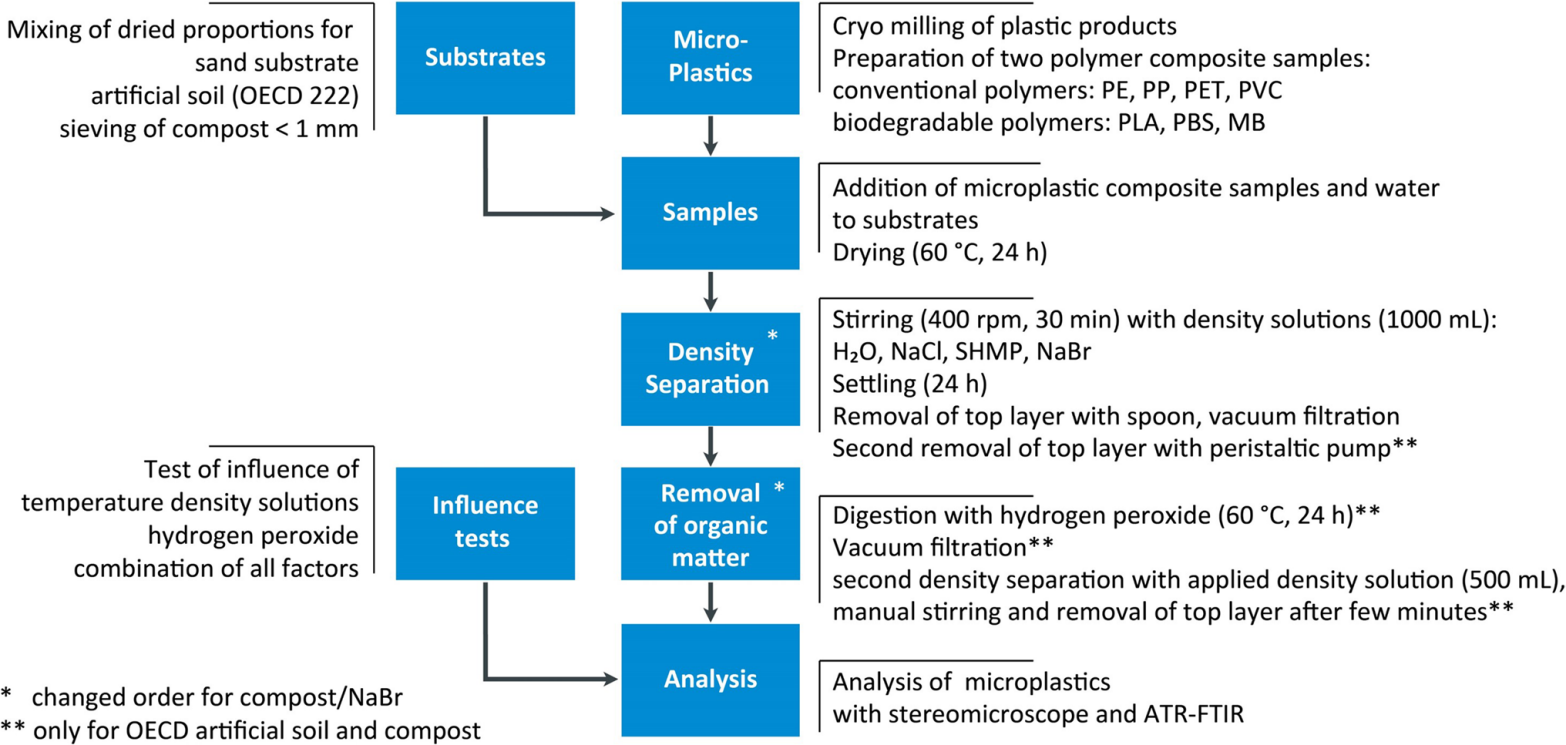
Flow chart of separation method

The experimental design to separate MPs from solid sample matrices included preparation of three different sample matrices and the addition of artificial MPs. Treatment steps of density separation and the digestion of organic matter were performed. Four different solutions were applied according to their density and toxicity. Recovery rates, as well as changes in size and surface structure of MPs, were analyzed with stereo microscope and Attenuated Total Reflectance–Fourier Transform Infrared spectroscopy (ATR-FTIR) (Fig. 1). A chemical identification of potential MPs is associated with the cost of an analyzer, but assumed to be mandatory, because of the high error rates of 20–70% through visual sorting (Bläsing and Amelung 2018). An ATR-FTIR analyzer is relatively cheap, e.g. compared to an automated µFPA-FTIR microscope. Therefore, 500 µm was chosen as the lowest size limit as it represents the optical resolution maximum of this technique.

To meet the standards of analytical quality assurance, every combination of plastic mixture, extraction solution and substrate was tested in triplicate with a blank for every series of tests to investigate contamination according to Quinn et al. (2017). Also, sources of MP contamination were excluded (see the “Contamination mitigation” section).

### Preparation of MPs

Different types of conventional and biodegradable plastic polymers were produced from mainly post-consumer products (Table 1). Conventional polymers were chosen according to their relevance and the probability of appearance as MPs in the environment. With polyethylene (PE) and polypropylene (PP) ($\rho =$ 0.862–1.083 g/cm^3^) as light density polymers, as well as polyvinyl chloride (PVC) and polyethylene terephthalate (PET) ($\rho =$ 1.286–1.369 g/cm^3^) as high-density polymers, it can be estimated whether the method is able to recover a variety of plastics with different densities. Densities were measured due displacement of volume with a pycnometer (50 mL) and ethanol (99%; TH. Geyer). Furthermore, three biodegradable polymers consisting of a range of newly produced polymers (PLA, PBS, MB) were applied for the first time in density separation validation tests from solid samples.

**Table 1:** Parameters of preparation of reference MPs

Polymer	Device	Speed [rpm]	Origin	Color	Density [g/cm^3^]
PE	Centrifugal mill (Retsch ZM 200)	8000	Pellet	Bright yellow	0.862
PP	Knife mill (Retsch GM 200)	6000	Pipe	Blue	1.083
	Centrifugal mill	6000			
PET	Scissor		Bottle	Green	1.216
	Centrifugal mill	6000			
PVC	Cutting		Pipe	Red	1.369
	Centrifugal mill	10,000			
PLA	Scissor Centrifugal mill	10,000	Coffee-to-go lid	White	1.300
PBS	Centrifugal mill	6000	Pellet	White	1.234
MB	Scissor Centrifugal mill	10,000	Biowaste bag	White-green	1.241

Reference MPs were prepared in four steps (Table 1): first, products were cut. Second, pieces were frozen in liquid nitrogen and ground (Elert et al. 2017). Third, size classes of 500–800 µm and 800–1000 µm were differentiated by sieving. Fourth, particles were visualized under a stereo microscope (Zeiss Stemi SV8 with Olympus SC30, f = 100, 80x, Oberkochen, Germany). Particles with a maximum length of 1.69 cm $\pm$ 0.40 cm and a minimum width of 1.08 cm $\pm$ 0.15 cm in the upper size class and a maximum length of 1.24 cm $\pm$ 0.33 cm and a minimum width of 0.77 cm $\pm$ 0.14 cm were gained with this method as the image analysis showed. All MPs were applied as fragments except MB, which was used as a film, because it is disposed as biowaste bags.

Two polymer composite samples were mixed, one with conventional and the other with biodegradable polymers. The tests for conventional polymers were conducted separately from the tests for biopolymers for easier observation. Concentrations of 400 particles/kg soil and 0.01% w/w, respectively, were concluded to reflect realistic concentrations of MPs in solid sample matrices and were also applied in other recovery tests (Liu et al. 2019). These assumptions resulted in a MPs count of 25 particles per polymer, consisting of 10 particles in the size range of 800–1000 µm and 15 particles between 500 and 800 µm. As it is likely that more small particles occur in soil (Braun et al. 2018), more particles of the smaller size class were applied. In detail, this means that we used 100 particles of the conventional polymer particles and 75 biopolymer particles in 250 g solid sample matrix each. The presence of biopolymers in solid sample matrices has not been investigated to date, but is estimated to be low. The number of particles used is based on the assumptions made for the conventional polymers (25 per type).

In addition to the densities of used MPs, the color, chemical spectra, as well as the sizes of particles before and after sample preparation, were determined (see the “Microscopy and FTIR polymer identification” section).

### Preparation of solid sample matrices

Three solid sample substrates (sand, OECD artificial soil, and compost) were examined, representing different environmental compartments (beach/sediment, terrestrial soil, and natural sample with high content of organic matter).

An amount of 250 g was used per sample. This is, together with Möller et al. (2021), the largest sample mass that has been investigated compared to previously published studies that measure MPs concentrations in soil with density separation.

### Sand substrates

Sands in different size classes ranging between 60–300 µm, 400–800 µm, and 700–1200 µm (Scherf Quarzsand), as well as silicon dioxide in a size range of 53–74 µm (Eijelkamp synthetic sand), were purchased to determine the influence of different particle sizes in the matrix. These were combined to sand sample substrates according to sand fractions in OECD artificial soil (Table 2). In relation to OECD 222, the size fraction of 0.05–0.2 mm has to make up at least 50%, which was evenly divided between the two obtained fractions in this section. The composition of the other 50% followed the formula of 100 µm $\equiv$ 5% of total substrate composition. Therefore, sand was dried at 200 °C overnight and sieved with a vibratory sieve shaker (Retsch AS 200) for 10 min, amplitude 1.2.

**Table 2:** Composition of OECD artificial soil and sand substrate

	Proportion [%]	Mass for sand substrate [g]	Mass for OECD artificial soil [g]	Size fraction [µm]	
	25	62.5	43.75	53–74	Sand
	25	62.5	43.75	80–200	Sand
	5	12.5	8.75	200–300	Sand
	10	25.0	17.50	300–500	Sand
	10	25.0	17.50	500–710	Sand
	5	12.5	8.75	710–800	Sand
	10	25.0	17.50	800–1000	Sand
	10	25.0	17.50	1000–1200	Sand
			35.00		Kaolinite
			15.00		Halloysite nanoclay/kaolin clay
			25.00		Sphagnum peat
Total mass [g]		250.00	250.00		

Afterwards, the sand sample substrates were placed in a muffle furnace at 550 °C for at least 6 h to remove potential MP contamination. Finally, the composite plastic samples were added to the sand sample substrates. The mixture was stirred manually for 30 s.

### OECD artificial soil

The OECD artificial soil is based on elaborations of an artificial soil in OECD 222: Earthworm Reproduction Test (*Eisenia fetida/Eisenia andrei*). This test was established for the ecotoxicological test of chemicals. It shows the advantage of creating a reproducible soil matrix with proportions of soil that can affect measurements of MPs through clay particles and soil organic matter (SOM) (Thomas et al. 2020).

The OECD guideline presets a content of 20% kaolin which was split between kaolinite (CAS No. 1318–74-7) and halloysite nanoclay/kaolin clay (CAS No. 1332–58-7) (Sigma-Aldrich). Kaolinite (Al_2_O_7_Si_2_ * 2H_2_O) and halloysite nanoclay/kaolin clay (Al_2_Si_2_O_5_(OH)_4_ * 2 H_2_O) are both 1:1-layered aluminosilicate minerals which belong to the kaolin group. The proportion of kaolinite (70%) was selected to be higher than of halloysite nanoclay/kaolin clay (30%) as it is the most widespread clay mineral in soils (Dixon 1989).

As there was no further definition of “finely ground,” the size of sphagnum peat (Floragard Floratorf) was oriented to the descriptions of OECD 232: Collembolan Reproduction Test in Soil which specifies finely ground to a particle size of 2 $\pm$ 1 mm. Therefore, sphagnum peat was sieved to 1.00–3.15 mm due to available mesh size.

All proportions were dried to constant weight.

A mixture of sand size classes with same proportions as the sand substrate was prepared (Table 2) and placed in a muffle furnace at 550 °C for at least 6 h. Afterwards, 35.00 g kaolinite and 15.00 g halloysite nanoclay/kaolin clay were added, as well as 25.00 g of sphagnum peat. Additionally, 1.25 g of CaCO_3_ were added, which corresponds to 0.5% of soil mass for obtaining pH 6.0  $\pm$ 0.5. The pH was determined according to OECD 222 by mixing 20 g of artificial soil with 100 mL KCl (1 M) thoroughly for 5 min. The mixture was left to settle for 2–4 h and then measured with a pH-meter (pH 526, WTW), calibrated at pH 4.0 and 7.0.

The formation of soil aggregates was accelerated by wetting and drying soil. Therefore, 45 mL of deionized water and the composite plastic sample were stirred together manually with the substrate for two minutes. Afterwards, 5–10 mL of deionized water were used to rinse the spoon. The sample was let stand for 4 days to create agglomerates with components and dried at 60 °C for 24 h. The applied amount of water was oriented to 60% of water holding capacity as demanded in OECD 222, which was assessed with HYPROP 2 (METER Group). Two samples of OECD artificial soil (250 g) were put in a sample ring and placed into water for complete saturation for 3 days. Afterwards, measurements were conducted over 13 days. The water holding capacity at pF 1.8 was calculated to 32.7%.

### Compost

Compost was derived from a biowaste plant in Lower Saxony, Germany (ALBA, Watenbüttel). The compost consists of biowaste from private households as well as garden waste (66%) and was fermented at > 55 °C for 14 days according to the composting plant operator. Characteristics show a content of organic matter triple the amount of OECD artificial soil. Furthermore, pollution with plastics is documented in the share of foreign matter with 0.08% dry weight. The moisture content of 38.5% was obtained by drying compost to a constant weight.

Compost was dried at 60 °C and sieved. The composite plastic sample and 100 mL of deionized water was added to 250 g of compost dry weight (size class < 1 mm) and stirred manually for 1 min to create agglomerates containing MPs, whereas the amount of added water corresponds to the moisture content before drying. Afterwards, samples were dried again (60 °C, 24 h). As compost was not newly mixed from components in contrast to OECD artificial soil, no standing time was applied.

### Preparation of density solutions

Density separation was performed with deionized water ($\rho =$ 1.00 g/cm^3^) and brine solutions of different densities. Saturated solutions were made by dissolving the respective salt into a volume of 1 L deionized water. In the case of NaCl (> 99%, z. A., Ph. Eur.; CHEMSOLUTE®) and NaBr (99%, p.a., ACS; Roth), the amount of salt per water volume (358 g NaCl /L H_2_O and 905 g NaBr/L H_2_O) was dependent on the specific solubility of each salt. The solutions were stirred in a beaker (2 L) on a magnetic stirring plate overnight, resulting in solution densities of ($\rho =$ 1.19 g/cm^3^) for NaCl and ($\rho =$ 1.53 g/cm^3^) for NaBr. As there was no information available about solubility of SHMP (65–70% P_2_O_5_ basis; Sigma-Aldrich), the substance was added to deionized water subsequently and stirred until no further salt dissolved and an excess formed at the beaker bottom ($\rho =$ 1.30 g/cm^3^).

To lower the amount of disposed chemicals as well as costs, separation solutions of SHMP and NaBr were recycled and reused throughout the test series of density separation. Solutions were decanted onto a pleated filter (Macherey–Nagel MN 615 1/4, diameter 385 mm). The salt was retrieved in a rotary evaporator (Rotavapor RE 111; Büchi Labortechnik AG). Nevertheless, solutions that were used for density separation with compost were not recycled as the dark color indicated pollution with humic substances from compost.

### Density separation, digestion, and recovery

The recovery rates of MPs in solid sample matrices were determined by density separation with deionized water and different salt solutions. Therefore, spiking experiments were performed, where 250 g of solid sample matrix was spiked with the composite plastic sample. First, 500 mL of the respective separation solution was added into a 2 L glass beaker and stirred with a rectangular stirring bar at 200 rpm. Afterwards, each spiked solid sample was transferred together with another 500 mL separation solution into the glass beaker, resulting in a total volume of 1 L separation solution per sample. Glass beakers were also used for density separation of MPs in soil in other studies (Chen et al. 2020; Huang et al. 2020; Liu et al. 2018; Zhou et al. 2020), as they are cheap, readily available, and easy to handle without the need to design complicated individual apparatus assemblies. Thus, they meet the requirement to be “available worldwide.”

Then, samples were stirred on a magnetic stirring plate at 400 rpm for 30 min to break agglomerates as MPs can be strongly sorbed to the matrix (Liu et al. 2018). Even though polymers were artificially added to solid samples in these test series, and it is hypothesized that strong sorption effects might not take place, it is likely that this time span is necessary when analyzing real soil samples. This hypothesis needs to be further tested. Afterwards, the stirring bar was removed and rinsed with the respective density solution as well as the beaker walls. Beakers were left to stand for about 24 h to allow density separation, chosen according to Hurley et al. (2018) and Liu et al. (2018). The settling time allows the MP particles to float up to the solution surface and heavier sample matrix particles, like inorganic contents, to sink to the beaker bottom.

The upfloating MPs were removed from the top layer of the solution surface with a spoon. This new method was applied because other methods, like decanting the supernatant, have difficulties like adhesion of MPs to container walls (Thomas et al. 2020). For sand substrates, MPs were then separated with tweezers, washed in deionized water, and stored in petri dishes. For OECD artificial soil and compost samples, organic matter was additionally surfaced to MPs. Remaining residues were collected by suction of a peristaltic pump in a second removal step (Masterflex Console Drive easyload 7518–00; Cole-Parmer GmbH; Wertheim; Germany) as suggested by Quinn et al. (2017). A glass tube connected to a hose was moved around the solution surface to collect floating MPs. The supernatant was filtered directly onto a filter (Whatman 589/1, diameter 125 mm) using a Buchner funnel and a vacuum pump. This two-stage process, in which most of the floating organic matrix first has to be removed with a spoon and only a small part of this matrix, in connection with the solution, has to be sucked off using a peristaltic pump, allows a minimal removal of the density separation solution and no clogging of the pump. Other practices applied in preliminary examinations, such as decanting or pouring, separated the density separation solution, but only minimally separated the floating organic residue. Thus, an easy to apply and time-saving two-step process could be developed. In comparison, other methods of removing the supernatant are performed up to four times (Zhang et al. 2018).

After removal from solution surface, the residues consisted mainly of organic matter. Mass ranged from 5 to 100 g depending on density solution and substrate which equals 2–40% of the total sample mass. Therefore, H_2_O_2_ (min. 30% w/w; CHEMSOLUTE®) was used as a reagent for oxidizing digestion of organic matter. It was successfully applied in many studies recovering MPs from solid sample matrices (e.g., Hurley et al. 2018; Han et al. 2019; Liu et al. 2018, 2019) and showed removal rates of 96–108% of SOM (Hurley et al. 2018).

The handling and the amount of the digestion solution had to be adapted depending on the substrate. The residues of compost samples were placed in a beaker (2 L) and H_2_O_2_ (30%) was added, depending on the amount of floating organic matter (ratio 1:4), and stirred at 50–60 rpm. The samples were heated for 24 h at 60 °C and let stand for 3 days at room temperature. Residues of OECD artificial soil samples were also heated at 60 °C for 24 h, but 100 mL H_2_O_2_ was not sufficient to cover the sample, so the digestion solution volume was increased to 200 mL H_2_O_2_ (30%) (ratio 1:8). Residues were filtered using a vacuum pump onto filters (Whatman 589/1, diameter 125 mm).

Afterwards, MPs were identified visually with the naked eye and under a stereo microscope and sorted out using tweezers. Added MPs were identified according to known colors and shapes. Films were considered according to lighter color and flexibility when touched with tweezers.

Then, a second density separation was applied. Residues were mixed with 500 mL of the respective density solution, stirred manually and let stand for 1–2 min. MPs were then sorted from the surface and washed in deionized water, dried at room temperature, and stored in petri dishes for analysis.

The treatment step that removes the most matrix should be applied first (Thomas 2020). Preliminary experiments showed that density separation eliminated most of the sample matrix compared to digestion. There was one exception in compost samples, with NaBr as density solution. Thus, density separation was applied before digestion in all experiments except compost with NaBr.

### Tests on influence of sample preparation

To test the influence of sample preparation on MPs, several potential impact factors were examined individually and also in combination. The individually tested impact factors were dry storage at 60 °C; applied separation solutions for density separation; H_2_O_2_ (30%) at room temperature, and H_2_O_2_ (30%) at 60 °C. Three particles per polymer type in a size > 1 mm were put into a petri dish and tested for 8 days for each impact factor except with H_2_O_2_ at 60 °C, which was tested for 3 days. The duration was based on the time periods of the separation process and corresponds to at least twice the time for each condition in the separation process described above. The combination was a sequence of dry storage at 60 °C, the separation solution NaBr that extracted the most MP contents and H_2_O_2_ at room temperature and at 60 °C with same durations as for individual factors, respectively. Afterwards, particles were washed in deionized water, dried and analyzed (see “Microscopy and FTIR polymer identification” section).

### Contamination mitigation

To prevent contamination of the samples with MPs during the ongoing experiments, cotton lab coats and butyronitrile gloves were worn in the laboratory and lab surfaces cleaned with deionized water (Quinn et al. 2017; Torres and De-la-Torre 2021). Equipment was cleaned in a dishwasher and dried in a drying oven. Furthermore, only filtered deionized water (20 $\upmu$ m) was applied in test series and for cleaning. All tests of density separation and removal of organic matter were performed under a flow bench and a fume hood. The time a sample was exposed to air was limited as much as possible to prevent atmospheric contamination. Additionally, beakers were covered with aluminum foil during stirring and settling. Furthermore, the air in the lab was filtered (Philips air purifier 2000 AC 2887; Koninklijke Philips N.V.; Amsterdam; Netherlands) as well as the supplied air from outside. Isolated MPs were stored in glass petri dishes until analysis. In addition, a blank sample was performed for each test series.

### Microscopy and FTIR polymer identification

MP composite samples and MPs for influence tests were manually sorted onto glass petri dishes and photographed under a stereo microscope (Zeiss Stemi SV8 with Olympus SC30, f = 100, 80x, Oberkochen, Germany) with AnalysisPro before and after application of the separation protocol.

Then, the photographs were evaluated by automatic computer image analysis to determine the damaging effects on the size of the MPs due to the impact factors. Image analysis was done using the open source computer vision library OpenCV (opencv.org): contours were localized with the openCV function findContours, and the particle area was calculated from the contour. Maximum particle length was identified by automatic stepwise contour rotation and the openCV function boundingRect. All pictures had identical scale (84 pixels/mm), and contours with an area smaller than 500 pixels (0,07 mm^2^) were regarded as noise and excluded from the processing. The particles were fragmented and not round which is more likely to reflect their occurrence in the environment. Some particles were also frayed. The fabricated MPs are multidimensional so a two-dimensional picture can only represent a certain aspect of the particles. Thus, two parameters of area and maximum length were considered to reduce effects of observational error.

Also, MPs were characterized chemically with ATR-FTIR (Bruker Tensor 27 with MVP-Pro, resolution 4 cm^−1^, 16 scans, area 4000–380 cm^−1^). To ensure sufficient visual classification, recovered MP from the composite MP samples were measured by ATR-FTIR. Due to the high particle number of 6300 used MPs, a factor of 25% used particle number was estimated. This led to the ATR-FTIR investigation of 25 conventional polymer particles and rounded up to 20 biopolymers per sample. For tests of influence, an analysis of changes in surface structure was performed with ATR-FTIR. Reference spectra were gained by accumulating six spectra of each polymer. For this, three particles per polymer were measured on two sides. After the tests of influence, the same procedure was applied for each polymer and impact factor and compared with reference spectra.

### Statistical analysis

For recovery tests, means, standard deviation, and recovery rates of MPs were calculated for each sample and compared for every combination of substrate and solution. Also, numbers of recovered MPs for the first and second removal from the solution surface were compared. For tests of influence, an analysis of changes in surface structure was performed with ATR-FTIR. The similarity of FTIR spectra was tested with Pearson’s correlation coefficient likewise to Vermeiren et al. (2020).

## Results and discussion

### Recovery rates of MPs

**Fig. 2:**
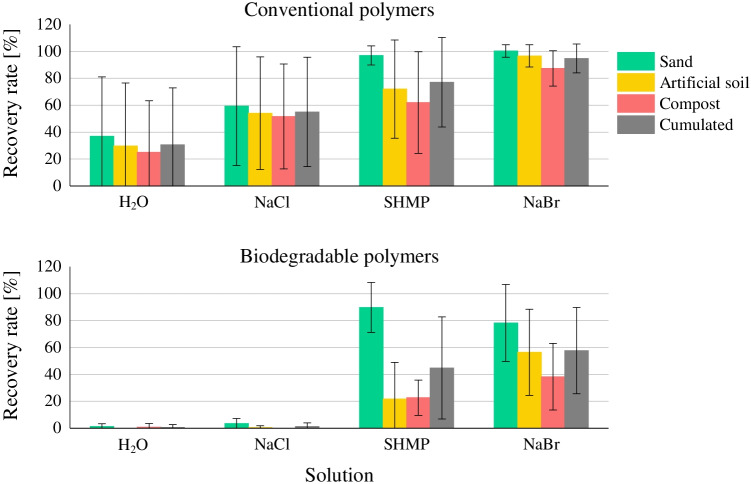
Recovery rates of conventional and biodegradable MPs depending on solution and substrate

Increasing densities of solutions were associated with increasing recovery rates of conventional and biodegradable MPs (Fig. 2). Similar results for conventional polymers were shown in other studies (Han et al. 2019; Vermeiren et al. 2020; Radford et al. 2021). Recovery rates were higher for conventional polymers than for biopolymers in every solution and substrate. As there were no other studies testing recovery rates of biodegradable polymers in soil, no comparisons can be made. It is assumed that the effect of sample treatment on size and shape of the biopolymers was low (see the “Changes in size of MPs from recovery tests” section). Therefore, visual sorting could be a limiting factor, so that the analytics might have to be expanded in this case. Recovery rates were, across all density solutions, the highest in following order: sand substrate > artificial soil > compost. This corresponds to the higher organic content.

In this scientific work, the highest conventional polymers were recovered with NaBr (94.8%$\pm$ 10.7%). Similar results with recovery rates > 90% were achieved in other studies, showing the comparability of the investigations made. MPs > 500 µm were recovered with 90.7% in ZnCl_2_ ($\rho =$ 1.5 g/cm^3^) in an organic rich sediment (Vermeiren et al. 2020). Another study with NaBr as density solution also recovered PE, PP, PET, and PVC with > 90% (Liu et al. 2019). In a solution of NaI with $\rho =$ 1.6 g/cm^3^, Zhou et al. (2020) recovered PVC with 75.8% and PE with 112.4% from soil (particle size 0.5–2 mm). In these test series, PVC showed even higher recovery rates (91.1% $\pm$ 12.8%) with NaBr. Therefore, due to the highest recovery rates of MPs in every substrate and simple handling in this study, the usage of NaBr can be recommended for further experiments. Additionally, for separation of low-density polymers PE and PP, application of NaCl as density solution is sufficient as recovery rates of 93.6% $\pm$ 8.5% illustrate. This justifies the application of NaCl as the most used salt for density separation of MPs from sediments (Cutroneo et al. 2021).

**Fig. 3:**
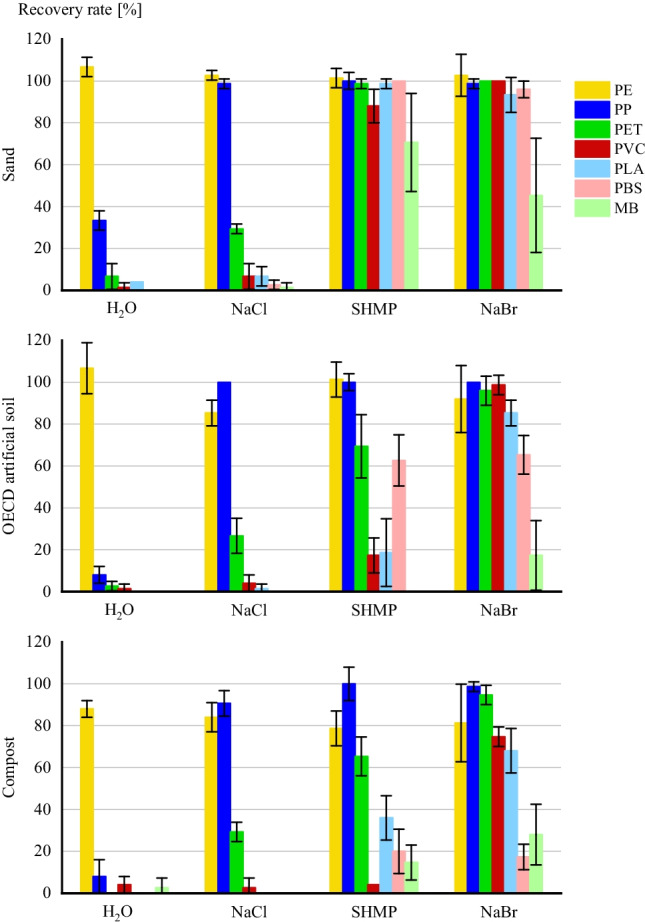
Recovery rates of MPs sorted by substrate and solution

Looking at recovery rates of different polymers, more polymer types were retrieved with increasing density of the applied solution (Fig. 3).

For sand substrates, PE was the only polymer that was recovered with > 90% in H_2_O. In another study with pure sand, PE and PP were applied in a similar concentration and retrieved with > 87% (Zhang et al. 2018) . Since the density of PP with $\rho =$ 0.91 g/cm^3^ was lower than in these investigations ($\rho =$ 1.08 g/cm^3^), density separation with water was able to achieve these results. Only PP and PE particles are recovered in NaCl with > 90%. PET and PVC show no sufficient retrieval with 18.0%  $\pm$ 13.1%, similar to other works (Liu et al. 2019; Han et al. 2019).

Recovery rates in SHMP and NaBr for conventional polymers are both high with higher recoveries of PVC in NaBr (100%  $\pm$ 0%) compared to SHMP (88.0% $\pm$ 8.0%) due to higher density of the solution. Biodegradable MPs were only retrieved in SHMP (89.8% $\pm$ 18.6%) and NaBr (78.2% $\pm$ 28.6%). In NaBr solution, salt crystals on beaker walls or solution surface hindered recovery. This was not observed in SHMP, accounting for higher recovery rates.

The lowest recovery rates in SHMP of 70.7% $\pm$ 23.4% and NaBr of 45.3% $\pm$ 27.2% are measured for MB which also showed the highest standard deviations. This can be attributed to the fact that MB films could not be sufficiently recovered due to the white-translucent color in liquids. Again, formation of salt crystals of NaBr interfered with identification. For example, to prevent salt crystal formation, the evaporation rate through cooling must be minimized over the period of sample treatment.

Matching results compared to sand substrate as for H_2_O can be observed in artificial soil with a similar recovery rate of 106.7% for PE. Comparable recovery rates in soil of > 90% of PE beads (850–1000 µm) with H_2_O were also measured by Hurley et al. (2018). However, recovery rates of PE were lower in other solutions (85–101%). PE particles were the hardest to recover of conventional polymers due to their color, which matched the brown-yellow color of residues of organic matter most closely. In addition, gas formation in second density separation hindered visual identification of particles on the solution surface in salt solutions. For this reason, except for PE, conventional polymers all showed the highest recovery rates in NaBr with > 92%.

Biodegradable MPs were also the highest recovered on NaBr with 17–85%. MB showed the lowest recovery which can be again attributed to decreased visibility. In the residues after digestion, only MB films with green color could be recovered. An effect of color on recovery rate was also described by Han et al. (2019) which showed the lowest recovery for PE due to same white color as mineral particles left in the sample. Liu et al. (2019) also recorded lower recovery rates of films compared to fibers and particles. The decreased visibility can also be a reason for lower recovery rates of PLA and PBS compared to conventional polymers.

There are greater differences for recovery rates with SHMP and NaBr in OECD artificial soil compared to sand substrate. PET and PVC showed notable higher recovery rates in sand substrate (NaBr: 97.3% $\pm$ 5.5%; SHMP: 43.3% $\pm$ 30.5%). The same was observed for PLA and PBS (NaBr: 76.0% $\pm$ 12.1%; SHMP: 32.7% $\pm$ 27.5%). These results cannot be explained by density of solution or the amount of floating organic matter as there were no differences between solutions which could have influenced recovery rates of MPs. It can only be speculated that the presence of organic matter in general, or kaolin, had an influence on the behavior of floating MPs. Higher standard deviations of MP concentrations in every OECD artificial soil sample for SHMP and NaBr compared to sand can be found relying on visual identification. This highlights the need to consider characteristics of soil organic matter when choosing a separation method (Thomas et al. 2020). Additionally, Radford et al. (2021) identified a decreased efficiency of removal of organic matter by H_2_O_2_ in the presence of SHMP and classified the solution as unsuitable. Similar effects could also be observed in these test series.

Recovery rates in compost samples show a matching trend to OECD artificial soil with increasing recovery rates in increasing density, but they were lower in every solution in compost compared to OECD artificial soil. The decreased recovery rates can be attributed to the content of organic matter which was also shown in other studies (Radford et al. 2021). Compost had a content of organic matter triple the amount of artificial soil. In addition, with the increasing density of solutions more organic matter of compost surfaced. Thus, more organic matter had to be filtered and digested after density separation. In addition, there were residues of compost left after digestion. Removal of organic matter showed varying efficiency, depending on applied density solution but also depending on sample in general. This is attributed to varying temperatures of heating plates with $\pm$ 5 °C and incomplete degradation of filters during digestion which hindered identification of polymers. For example, similar to artificial soil, MB particles could only be recovered when having a green color, resulting in recovery rates < 30%.

As mentioned before, the sequence of density separation and removal of organic matter was changed for compost samples with NaBr solutions as preliminary tests revealed surfacing of 80–90% of the sample. Based on research findings by Hurley et al. (2018), it was assumed that there is no considerable effect of the sequence on efficiency of separation of MPs. However, samples were then easier to handle. Since the whole sample was not digested, residues of organic matter also surfacing in density separation.

No MPs greater than 500 µm were detected in blanks of artificial soil and sand and no intentionally added MPs in blanks of compost.

A disadvantage of the method is that it currently relies on visual sorting from solution surface as well as from filters after removal of organic matter and is therefore only applicable for identification of MPs > 500 µm. Separation of residues of organic matter from the sample has to be increased to make an automated analysis of smaller MPs possible.

Depending on sample and density solution, different amounts of organic matter were flooded. In artificial soil, the whole amount of organic matter surfaced in all test series while in compost, it depended on the density of solution. This depicts a different behavior of organic matter based on the type and composition. Thus, the application of different methods to remove organic matter can be helpful. To increase recovery rates and decrease visual dependency, conditions of digestion have to be improved to digest organic matter completely like increase heating to 24 h or using more reagent. Otherwise, a second digestion after density separation can be implemented.

Considering the recovery rates of the second removal from solution surface, 1–3% of all recovered particles were recovered in second removal step with the same density solution, depending on the substrate (Table 3). Thus, this step is not concluded to be necessary for further studies, when applying the described extraction method. However, for other extraction methods, several extraction steps are recommended (Zhang et al. 2018). Unfortunately, these data were not collected in other further studies although several extraction steps were performed (Chen et al. 2020; Huang et al. 2020).

**Table 3:** MPs recovered due to second removal of solution surface

Substrate	Recovered particles	Share of recovered particles [%]	Share of affected samples [%]
Artificial soil	9	1.0	6.0
Compost	25	3.0	15.5

### Impact of sample preparation on MP size

Similar to Liu et al. (2019), the impact of H_2_O was set as a reference point indicating no impact of sample preparation. Changes in size of polymers varied $\pm$ 2% for both parameters (area and maximum length) with H_2_O which suggests this as an observational error. All impact factors except H_2_O_2_ at 60 °C showed only changes of size in this margin of uncertainty (Table 4).

**Table 4:** Changes in size of polymer particles due to influence factors. Two parameters of area and maximum length were compared. Changes above observational error of $\pm$ 2% are bold

Influence factor	Polymer	Average area before tests (cm^2^)	Average area after (cm^2^)	Difference in area (cm^2^)	Maximum length before tests (cm)	Maximum length after tests (cm)	Difference in maximum length (cm)	Difference in area (%)	Difference in maximum length (%)
H_2_O	PE	1.85	1.68	0.17	2.91	2.86	0.05	**9.16**	1.79
	PP	3.14	3.09	0.05	2.87	2.88	0.00	1.54	− 0.14
	PET	4.69	4.68	0.01	3.51	3.53	− 0.02	0.15	− 0.69
	PVC	2.42	2.42	0.00	2.05	2.07	− 0.02	0.11	− 0.78
	PLA	1.89	1.91	− 0.01	2.33	2.33	0.00	− 0.70	− 0.17
	PBS	2.09	2.04	0.05	2.06	2.04	0.02	**2.17**	0.78
	MB	4.88	4.84	0.04	3.80	3.71	0.08	0.75	**2.12**
NaCl	PE	1.64	1.65	− 0.01	1.90	1.86	0.03	− 0.44	1.70
	PP	2.13	2.06	0.07	2.22	2.20	0.02	**3.39**	0.91
	PET	4.13	4.10	0.03	3.07	3.20	− 0.12	0.76	− 4.05
	PVC	1.95	1.88	0.07	1.96	1.89	0.07	**3.77**	**3.69**
	PLA	1.96	1.94	0.01	2.05	2.04	0.01	0.77	0.59
	PBS	2.29	2.26	0.03	2.09	2.06	0.02	1.48	1.15
	MB	2.80	2.71	0.08	3.25	3.18	0.06	**3.04**	1.86
SHMP	PE	2.10	2.13	− 0.03	2.08	2.07	0.00	− 1.29	0.19
	PP	2.68	2.69	− 0.01	2.49	2.51	− 0.02	− 0.36	− 0.97
	PET	4.02	3.93	0.10	3.99	3.80	0.19	**2.47**	**4.73**
	PVC	2.33	2.34	− 0.01	2.10	2.08	0.02	− 0.32	0.95
	PLA	3.17	3.16	0.00	2.85	2.89	− 0.04	0.16	− 1.27
	PBS	1.82	1.83	− 0.01	1.87	1.84	0.03	− 0.77	1.50
	MB	2.44	2.33	0.11	2.73	2.66	0.07	**4.36**	**2.50**
NaBr	PE	2.19	2.14	0.05	2.10	2.08	0.02	**2.21**	1.15
	PP	2.88	2.89	− 0.01	2.58	2.57	0.01	− 0.32	0.47
	PET	3.95	3.88	0.07	2.90	2.90	− 0.01	1.78	− 0.28
	PVC	2.26	2.30	− 0.04	2.12	2.14	− 0.02	− 1.68	− 0.95
	PLA	2.10	2.12	− 0.02	2.13	2.14	− 0.02	− 0.90	− 0.75
	PBS	1.91	1.93	− 0.02	2.02	2.00	0.02	− 1.25	0.99
	MB	3.39	3.45	− 0.06	3.73	3.16	0.57	− 1.90	**15.19**
Temperature of 60 °C	PE	1.83	1.77	0.06	2.14	2.06	0.08	**3.25**	**3.57**
	PP	2.47	2.39	0.08	2.62	2.55	0.07	**3.23**	**2.76**
	PET	6.81	5.53	1.28	4.22	3.53	0.69	**18.77**	**16.37**
	PVC	2.27	1.99	0.28	1.95	1.86	0.09	**12.46**	**4.73**
	PLA	1.92	1.91	0.01	2.25	2.22	0.03	0.63	1.25
	PBS	1.47	1.48	− 0.01	1.67	1.67	− 0.01	− 0.35	− 0.48
	MB	2.89	2.99	− 0.10	2.80	2.88	− 0.07	− 3.33	− 2.58
H_2_O_2_ at RT	PE	1.91	1.97	− 0.06	1.99	2.06	− 0.07	− 3.20	− 3.43
	PP	2.20	2.12	0.08	2.05	2.05	0.00	**3.66**	0.00
	PET	5.55	5.44	0.11	3.53	3.56	− 0.03	**2.04**	− 0.91
	PVC	2.28	2.30	− 0.02	2.12	2.12	0.01	− 0.82	0.38
	PLA	1.56	1.55	0.01	1.82	1.82	0.00	0.57	− 0.22
	PBS	1.91	1.90	0.01	2.03	2.00	0.03	0.49	1.38
	MB	3.45	3.33	0.12	3.33	3.29	0.04	**3.39**	1.09
H_2_O_2_ at 60 °C	PE	1.62	1.67	− 0.05	1.84	2.03	− 0.19	− 2.91	− 10.24
	PP	3.41	3.45	− 0.03	2.66	2.69	− 0.03	− 0.97	− 1.06
	PET	4.56	4.63	− 0.06	3.39	3.41	− 0.01	− 1.38	− 0.35
	PVC	1.96	1.88	0.07	1.94	1.90	0.04	**3.67**	**2.07**
	PLA	2.18	2.15	0.03	2.19	2.20	− 0.01	1.33	− 0.55
	PBS	2.27	2.27	0.00	2.20	2.16	0.04	− 0.13	1.83
	MB	3.07	2.24	0.83	3.13	2.75	0.38	**27.11**	**12.19**
Combination of factors	PE	1.88	1.86	0.01	1.94	1.93	0.01	0.75	0.62
	PP	2.88	2.87	0.00	2.47	2.44	0.03	0.17	1.14
	PET	4.04	5.10	− 1.06	3.04	3.02	0.02	− 26.25	0.66
	PVC	2.20	2.31	− 0.10	2.13	2.18	− 0.05	− 4.71	− 2.26
	PLA	2.16	2.13	0.03	2.31	2.37	− 0.06	1.26	− 2.60
	PBS	3.45	3.34	0.11	2.69	2.58	0.11	**3.11**	**4.03**
	MB	3.87	3.34	0.53	3.72	3.16	0.56	**13.67**	**15.10**

**Fig. 4:**
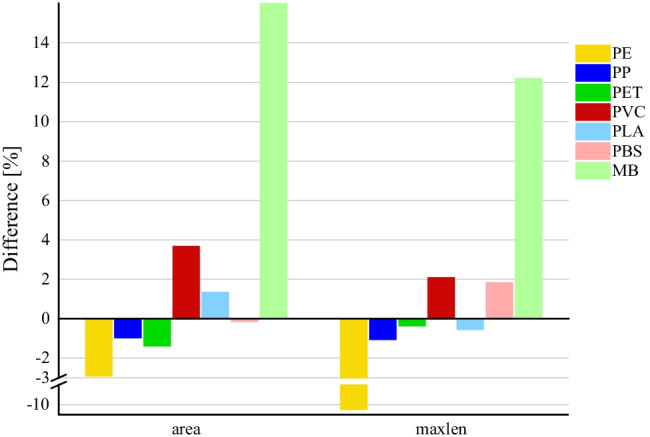
Decrease in size due to influence of H_2_O_2_ at 60 °C

**Fig. 5:**
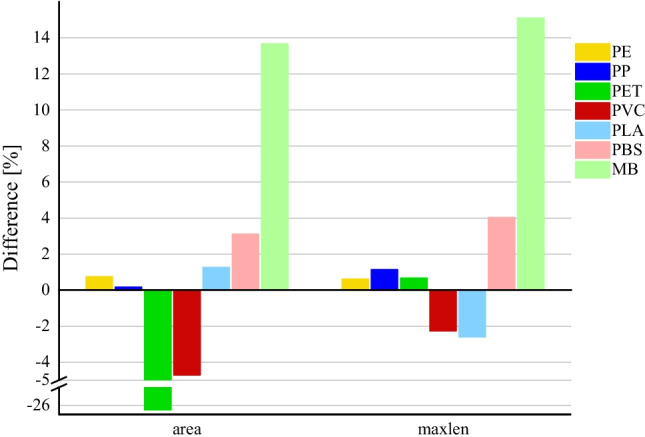
Decrease in size due to influence of combination of factors (temperature of 60 °C; NaBr; H_2_O_2_ at room temperature and at 60 °C)

**Fig. 6:**
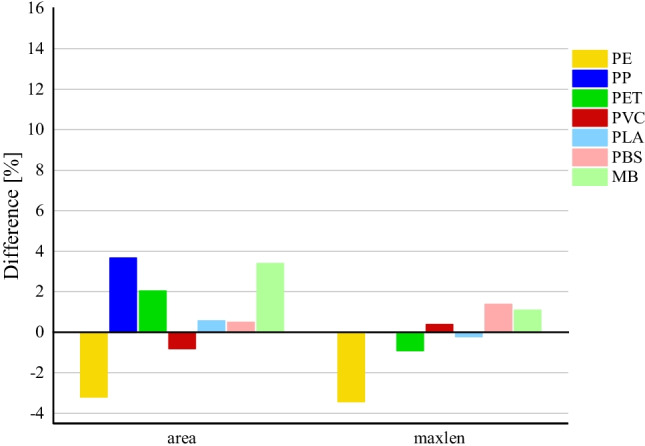
Decrease in size due to influence of H_2_O_2_ at RT

With H_2_O_2_ at 60 °C, MB particles decreased by 12.2–27.1% (Fig. 4). This is also reflected in the decrease of size of MB particles with the combination of impact factors (temperature of 60 °C; NaBr; H_2_O_2_ at room temperature and at 60 °C) (Fig. 5). At room temperature, no effects on MPs by H_2_O_2_ were shown considering the preconditions (Fig. 6). The same conditions of H_2_O_2_ at 60 °C for 3 days were applied by Liu et al. (2019). No considerable changes of conventional polymers were shown (these were defined > 10%). The same applies for considered conventional polymers in tests of Hurley et al. (2018) with a time period of 24 h.

For PET particles in tests of combination of impact factors, a substantial increase in area of + 26% was measured although no change in maximum length was detected. In addition, this effect was not recorded for other impact factors. No specific cause could be identified.

### Spectral changes of polymer structures

**Fig. 7:**
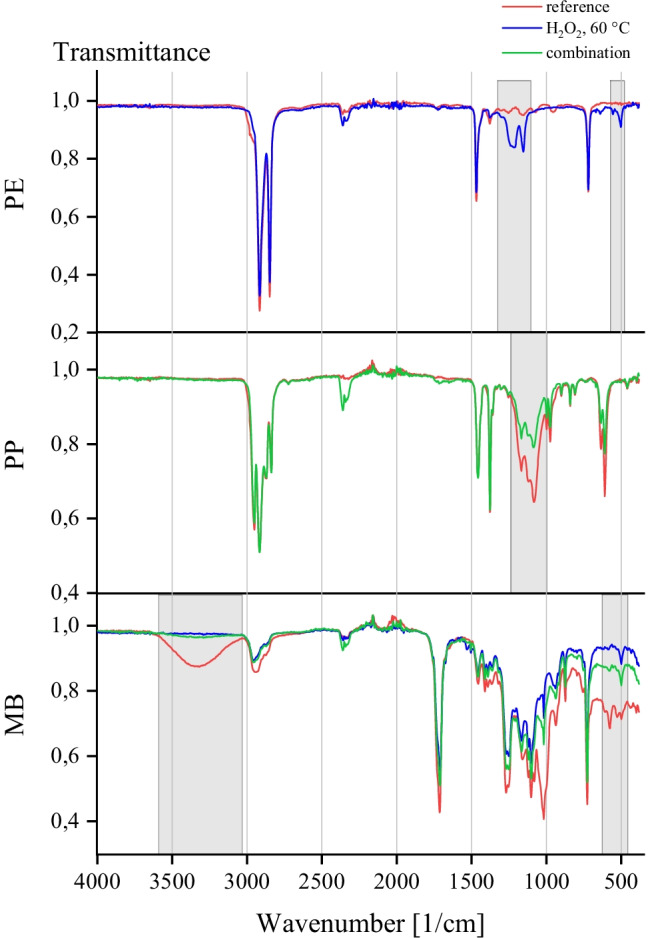
Comparison of IR-spectra of PE, PP and MB with Pearson’s correlation coefficients > 0.98 compared to reference. Areas with visible differences are marked

The comparison of spectra of impact factors with reference spectra reveals changes for PE, PP, and MB in H_2_O_2_ at 60 °C as well as combination of factors based on Pearson’s correlation coefficients > 0.98 (Fig. 7).

There are two additional peaks at a wavelength between 1213 and 1153 cm^−1^ for MPs with H_2_O_2_ at 60 °C as impact factor. These could be attributed to C–O bonding (Hesse et al. 2012) and showed an oxidation of molecules at the surface of particles. It is likely that more radicals of H_2_O_2_ are produced at a higher temperature which increases reactivity. It was shown that oxidation of PE can occur in concentrated solutions of H_2_O_2_ and at an elevated temperature, (Saunders 1988). Abiotic oxidation is also the first step for degradation in the environment (Gewert et al. 2015). Another peak at 503 cm^−1^ could not be allocated due to missing information about this wavelength (Hesse et al. 2012). Nevertheless, there is no effect visible in the spectrum of the test series with combination of impact factors. Despite same settings, it could be observed during experiments that temperatures of heating plates varied $\pm$ 5 °C which could have influenced changes in this case.

For MB, a peak at 3317 cm^−1^ is missing in the polymer spectra for H_2_O_2_ at 60 °C and combination of factors which can be attributed to changes of hydrogen bonds (Hesse et al. 2012). Furthermore, there are differences in the intensity of transmittance for MB due to the method of fixation in the ATR-FTIR.

The same approach was conducted with H_2_O_2_ at room temperature by (Vermeiren et al. 2020) with similar results of Pearson’s correlation coefficient > 0.900 for considered conventional polymers. Across all measured conventional polymers, correlation coefficients with 0.931 $\pm$ 0.025 were detected which is lower than in these experiments with 0.996  $\pm$ 0.003, so effects on polymer bonds were to a lesser extent. IR-spectra of conventional polymers with H_2_O_2_ at 60 °C also showed “no major deviations from control” (Hurley et al. 2018). Effects of H_2_O_2_ on MPs were also not detected by Radford et al. (2021).

The results of spectral changes fit results of size change for MB with H_2_O_2_ at 60 °C as impact factor. However, oxidation of PE was not visible in measuring of size in tests of influence. All in all, influence tests revealed that application of H_2_O_2_ at 60 °C showed a decrease of size of MB and changes in hydrogen bonds as well as oxidizing effects at the surface of PE.

Thus, it is concluded that lower recovery rates of biodegradable polymers mainly depend on appearance of polymers with reduced visibility during analysis of samples and not on chemical properties of the polymer.

For further studies, experiments of removal of organic matter with Fenton’s reagent are recommended. With the same efficiency, temperatures could be decreased which can decrease effects on particles (Hurley et al. 2018).

### Changes in size of MPs from recovery tests

Compared to previously described tests of influence, there is additional mechanical stress on MPs. It is assumed that the sample preparation using a cryomill for the production of the polymer reference material, in particular PE, caused predetermined breaking points in the material. These brittle MPs were affected in size by further mechanical stress from the processing methods, e.g. stirring. It is therefore noted that the quality of the reference material must be ensured for tests to determine the influence of the method on the polymer size. The use of the cryomill for the production of microplastic reference materials would have to be validated in further studies. However, due to the mainly round polymer shape, commercially available microplastics can only be compared to a limited extent with naturally occurring fragmented MP particles, e.g., with regard to soil aggregates. Nonetheless, the use of cryo-ground MP is recommended for determination of recovery rates. A change in size < 3.8% was regarded as observational error as this was the highest alleged growth of particles. Only means of polymers with recovery rates $\ge$ 100% were compared, which concerned 51 polymer samples in total. Considering area and maximum length, only particles of PE in OECD artificial soil show decreases in size with 5.1–20.4% (Table 5). Combined with the results of tests of influence, the decrease in size can be attributed to mechanical stress during density separation by stirring rather than the polymer itself. This is in coherence with tests of recovery where it was shown that several PE particles were destroyed. Because PE particles were brittle compared to other polymer particles, they were more easily destroyed. If the polymer type were the reason, this would also have been visible in the tests of influence. Thus, materials like separatory funnels and spiral conveyors which are not compressing particles between stirrer and vessel should be reconsidered (Enders et al. 2020; Möller et al. 2021).

**Table 5:** Decrease in size of recovered particles with recovery rates > 100% per sample. Decreases in size > 3.8% are bold. In rows without standard deviation, only one sample showed recovery rates > 100%

Polymer	Substrate	Solution	maxlen [%]	Area [%]
PE	Sand	H2O	3.4 $\pm$ 3.0	**5.6 **$\pm$** 4.5**
PE	Artificial soil	H2O	**17.3 **$\pm$** 3.8**	**20.4 **$\pm$** 12.8**
PE	Sand	NaCl	0.7 $\pm$ 2.5	− 0.9 $\pm$ 4.9
PE	Sand	SHMP	**6.7 **$\pm$** 6.9**	3.1 $\pm$ 5.7
PE	Artificial soil	SHMP	**7.4 **$\pm$** 0.3**	**13.7 **$\pm$** 4.7**
PE	Sand	NaBr	3.5 $\pm$ 2.9	**4.2 **$\pm$** 1.1**
PE	Artificial soil	NaBr	**5.1**	**13.4**
PP	Sand	NaCl	− 2.5 $\pm$ 2.9	− 1.7 $\pm$ 2.8
PP	Artificial soil	NaCl	− 0.4 $\pm$ 0.4	0.7 $\pm$ 1.3
PP	Sand	SHMP	− 0.3 $\pm$ 1.5	1.3 $\pm$ 0.5
PP	Artificial soil	SHMP	3.0 $\pm$ 3.9	1.9 $\pm$ 7.2
PP	Compost	SHMP	0.3 $\pm$ 2.6	3.0 $\pm$ 0.2
PP	Sand	NaBr	− 1.1 $\pm$ 0.7	− 3.8 $\pm$ 0.7
PP	Artificial soil	NaBr	0.3 $\pm$ 5.0	1.5 $\pm$ 6.3
PP	Compost	NaBr	3.4 $\pm$ 0.7	**10.3 **$\pm$** 2.7**
PET	Sand	SHMP	1.1 $\pm$ 0.2	1.3 $\pm$ 0.2
PET	Sand	NaBr	0.9 $\pm$ 2.0	− 1.8 $\pm$ 2.2
PET	Artificial soil	NaBr	1.8 $\pm$ 1.3	**4.0 **$\pm$** 3.7**
PET	Compost	NaBr	2.4	**8.1**
PVC	Sand	NaBr	− 0.9 $\pm$ 1.0	− 2.2 $\pm$ 2.6
PVC	Artificial soil	NaBr	3.2	2.3
PLA	Sand	NaBr	− 1.7	− 2.6
PBS	Sand	SHMP	0.7 $\pm$ 1.2	− 0.4 $\pm$ 1.3
PBS	Sand	NaBr	− 0.4	0.5

Polymer samples with < 100% recovery rate were not included as changes in size are then mainly influenced by the size of recovered MPs. This concerned 201 polymer samples. For this reason, no measurements of MB could be conducted and results of influence tests not be proven. However, the evaluation method cannot exclude the possibility that there is also an effect on the other polymers.

## Conclusion

There is a need for standardized procedures for MPs analysis to collect data for a true picture of the scale of MP contamination of the global environment. Thus, methods should be applicable around the globe. The introduced protocol provides a simple and useful tool for separation of conventional and biodegradable MPs from solid sample matrices with different composition representing different environmental compartments (beach/sediment, terrestrial soil, and natural sample with high content of organic matter), which are supposed to be sinks for MPs. It was shown that recovery rates of MPs increase with increasing density of separation solution and decrease with increasing content of organic matter within the sample matrix. To isolate a wide range of environmentally relevant polymers, solutions with a density > 1.5 g/cm^3^ are recommended.

This study showed recovery rates of 94.7% $\pm$ 10% with NaBr as density solution for conventional polymers with a density of 0.86–1.37 g/cm^3^ with an inexpensive, easy set up for density separation with a salt solution that does not harm the environment and has minimal effect on particle size and composition. Concentrations of 0.01% w/w MPs in solid matrices were recovered which shows applicability for realistic concentrations in solid matrices.

Indeed, it could be shown that the method needs to be optimized for isolating biodegradable polymers. This can be mainly attributed to the decreased visibility of the applied particles. In follow-up tests, it is crucial to reduce the dependence on visibility of the method. However, this is the first study to isolate biodegradable MPs from solid sample matrices. Although the share of bioplastics from globally produced plastics is currently < 1%, it is expected to grow. Thus, it is important to further investigate formation and quantification of MPs from bioplastics in solid sample matrices.

With a size limit of 500 µm and the set up with beakers, the method applies as a common minimum consensus. To get a full picture of MP contamination with respect to particles < 500 µm, the procedure has to be specialized on the sample matrix. Furthermore, it is possible that the method needs to be adjusted depending on the soil type e.g. to break agglomerates. As the set up possibly leads a higher effort for sample isolation and causes misidentifications, more specialized apparatus, such as separating funnels, are recommended. In addition, more elaborate and expensive analytical methods have to be applied.

Nevertheless, the presented method offers a simple and inexpensive way to isolate larger MP particles (> 500 µm) from different solid sample matrices and shows first results regarding sample treatment for biopolymer analysis.

## Data Availability

The datasets used and/or analyzed during the current study are available from the corresponding author on reasonable request.

## References

[CR1] Barceló D, Picó Y (2019). Microplastics in the global aquatic environment: analysis effects, remediation and policy solutions. J Environ Chem Eng.

[CR2] Bläsing M, Amelung W (2018). Plastics in soil: analytical methods and possible sources. Sci Total Environ.

[CR3] Boots B, Russell CW, Green DS (2019). Effects of microplastics in soil ecosystems: above and below ground. Environ Sci Technol.

[CR4] Braun U, Jekel M, Gerdts G, et al. (2018) Mikroplastik-Analytik: Probenahme, Probenaufbereitung und Detektionsverfahren: Diskussionspapier im Rahmen des Forschungsschwerpunktes Plastik in der Umwelt. Bundesministerium für Bildung und Forschung.

[CR5] Büks F, Kaupenjohann M (2020). Global concentrations of microplastic in soils a review. SOIL.

[CR6] Cerli C, Celi L, Kalbitz K, Guggenberger G, Kaiser K (2012). Separation of light and heavy organic matter fractions in soil testing for proper density cut-off and dispersion level. Geoderma.

[CR7] Chen Y, Leng Y, Liu X, Wang J (2020). Microplastic pollution in vegetable farmlands of suburb Wuhan, central China. Environ Pollut.

[CR8] Chubarenko I, Bagaev A, Zobkov M, Esiukova E (2016). On some physical and dynamical properties of microplastic particles in marine environment. Mar Pollut Bull.

[CR9] Corradini F, Meza P, Eguiluz R, Casado F, Huerta-Lwanga E, Geissen V (2019). Evidence of microplastic accumulation in agricultural soils from sewage sludge disposal. Sci Total Environ.

[CR10] Cutroneo L, Reboa A, Geneselli I, Capello M (2021). Considerations on salts used for density separation in the extraction of microplastics from sediments. Mar Pollut Bull.

[CR11] de Souza Machado AA, Kloas W, Zarfl C, Hempel S, Rillig MC (2018). Microplastics as an emerging threat to terrestrial ecosystems. Glob Chang Biol.

[CR12] Ding L, Zhang S, Wang X (2020). The occurrence and distribution characteristics of microplastics in the agricultural soils of Shaanxi Province, in north-western China. Sci Total Environ.

[CR13] Dixon JB (1989). Kaolin and serpentine group minerals. In Minerals in Soil Environments (eds J.B. Dixon and S.B. Weed). 10.2136/sssabookser1.2ed.c10

[CR14] Elert AM, Becker R, Duemichen E, Eisentraut P, Falkenhagen J, Sturm H, Braun U (2017). Comparison of different methods for MP detection: what can we learn from them, and why asking the right question before measurements matters?. Environ Pollut.

[CR15] Enders K, Lenz R, do IvarSul JA, Tagg AS, Labrenz M (2020). When every particle matters: a QuEChERS approach to extract microplastics from environmental samples. MethodsX.

[CR16] European Bioplastics (2019) Bioplastics market data 2019. https://docs.european-bioplastics.org/publications/market_data/Report_Bioplastics_Market_Data_2019.pdf*. *Accessed 15 August 2021

[CR17] Fan Y, Zheng K, Zhu Z, Chen G, Peng X (2019). Distribution, sedimentary record, and persistence of microplastics in the Pearl River catchment, China. Environ Pollut.

[CR18] Fuller S, Gautam A (2016). A procedure for measuring microplastics using pressurized fluid extraction. Environ Sci Technol.

[CR19] Gewert B, Plassmann MM, MacLeod M (2015). Pathways for degradation of plastic polymers floating in the marine environment. Environ Sci Process Impacts.

[CR20] Geyer R, Jambeck JR, Law KL (2017). Production, use, and fate of all plastics ever made. Sci Adv.

[CR21] Guo J-J, Huang X-P, Xiang L (2020). Source migration and toxicology of microplastics in soil. Environ Int.

[CR22] Haider TP, Völker C, Kramm J, Landfester K, Wurm FR (2019). Plastics of the future? The impact of biodegradable polymers on the environment and on society. Chem Int Ed.

[CR23] Han X, Lu X, Vogt RD (2019). An optimized density-based approach for extracting microplastics from soil and sediment samples. Environ Pollut.

[CR24] Hartmann NB, Hüffer T, Thompson RC (2019). Are we speaking the same language? Recommendations for a definition and categorization framework for plastic debris. Environ Sci Technol.

[CR25] Hesse M, Meier H, Zeeh B (2012). Spektroskopische Methoden in der organischen Chemie: 114 Tabellen.

[CR26] Huang Y, Liu Q, Jia W, Yan C, Wang J (2020). Agricultural plastic mulching as a source of microplastics in the terrestrial environment. Environ Pollut.

[CR27] Huerta Lwanga E, Vega JM, Quej VK (2017). Field evidence for transfer of plastic debris along a terrestrial food chain. Sci Rep.

[CR28] Hurley RR, Lusher AL, Olsen M, Nizzetto L (2018). Validation of a method for extracting microplastics from complex, organic-rich, environmental matrices. Environ Sci Technol.

[CR29] Lambert S, Wagner M (2017). Environmental performance of bio-based and biodegradable plastics: the road ahead. Chem Soc Rev.

[CR30] Liao J, Chen Q (2021). Biodegradable plastics in the air and soil environment: Low degradation rate and high microplastics formation. J Hazard Mater.

[CR31] Liu M, Lu S, Song Y (2018). Microplastic and mesoplastic pollution in farmland soils in suburbs of Shanghai, China. Environ Pollut.

[CR32] Liu M, Song Y, Lu S (2019). A method for extracting soil microplastics through circulation of sodium bromide solutions. Sci Total Environ.

[CR33] Möller JN, Heisel I, Satzger A, et al. (2021) Tackling the challenge of extracting microplastics from soils: a protocol to purify soil samples for spectroscopic analysis. Environ Toxicol Chem10.1002/etc.5024 10.1002/etc.5024 33620097

[CR34] Morra L, Bilotto M, Cerrato D (2016). The Mater-Bi biodegradable film for strawberry (Fragaria x ananassa Duch) mulching: effects on fruit yield and quality. Ital J Agron.

[CR35] O’Kelly BC, El-Zein A, Liu X, et al. (2021) Microplastics in soils: an environmental geotechnics perspective. Environ Geotech 1–3310.1680/jenge.20.00179

[CR36] OECD 222 (2016): Earthworm Reproduction Test (Eisenia fetida/ Eisenia andrei). Organisation for Economic Co-operation and Development.

[CR37] Piehl S, Leibner A, Löder MGJ, Dris R, Bogner C, Laforsch C (2018). Identification and quantification of macro- and microplastics on an agricultural farmland. Sci Rep.

[CR38] PlasticsEurope (2020) Plastics - the facts 2020: an analysis of European plastics production, demand and waste data. https://www.plasticseurope.org/application/files/8016/1125/2189/AF_Plastics_the_facts-WEB-2020-ING_FINAL.pdf. Accessed 15 August 2021

[CR39] Qin M, Chen C, Song B (2021). A review of biodegradable plastics to biodegradable microplastics: another ecological threat to soil environments?. J Clean Prod.

[CR40] Quinn B, Murphy F, Ewins C (2017). Validation of density separation for the rapid recovery of microplastics from sediment. Anal Methods.

[CR41] Radford F, Zapata-Restrepo LM, Horton AA, Hudson MD, Shaw PJ, Williams ID (2021). Developing a systematic method for extraction of microplastics in soils. Anal Methods.

[CR42] Rillig MC, Ingraffia R, de Souza M, Anderson A (2017). Microplastic incorporation into soil in agroecosystems. Front Plant Sci.

[CR43] Saunders KJ (1988) Organic polymer chemistry: An introd. to the organ. chemistry of adhesives, fibres, paints, plastics and rubbers, 2. ed. Chapman and Hall, London

[CR44] Schell T, Rico A, Vighi M (2020) Occurrence fate and fluxes of plastics and microplastics in terrestrial and freshwater ecosystems. In: Reviews of Environmental Contamination and Toxicology. Springer New York 10.1007/398_2019_40 32025906

[CR45] Scheurer M, Bigalke M (2018). Microplastics in Swiss floodplain soils. Environ Sci Technol.

[CR46] Thakur S, Chaudhary J, Sharma B (2018). Sustainability of bioplastics: opportunities and challenges. Curr Opin Green Sustain Chem.

[CR47] Thomas D, Schütze B, Heinze WM, Steinmetz Z (2020). Sample preparation techniques for the analysis of microplastics in soil a review. Sustainability.

[CR48] Torres FG, De-la-Torre GE (2021). Historical microplastic records in marine sediments: current progress and methodological evaluation. Reg Stud Mar Sci.

[CR49] van den Berg P, Huerta-Lwanga E, Corradini F, Geissen V (2020). Sewage sludge application as a vehicle for microplastics in eastern Spanish agricultural soils. Environ Pollut.

[CR50] Vermaire JC, Pomeroy C, Herczegh SM, Haggart O, Murphy M (2017). Microplastic abundance and distribution in the open water and sediment of the Ottawa River, Canada, and its tributaries. FACETS.

[CR51] Vermeiren P, Muñoz C, Ikejima K (2020). Microplastic identification and quantification from organic rich sediments: a validated laboratory protocol. Environ Pollut.

[CR52] Wang Q, Adams CA, Wang F, Sun Y, Zhang S (2021) Interactions between microplastics and soil fauna: a critical review. Crit Rev Environ Sci Technol 1–3310.1080/10643389.2021.1915035

[CR53] Zhang S, Yang X, Gertsen H, Peters P, Salanki T, Geissen V (2018). A simple method for the extraction and identification of light density microplastics from soil. Sci Total Environ.

[CR54] Zhang X, Li Y, Ouyang D (2021). Systematical review of interactions between microplastics and microorganisms in the soil environment. J Hazard Mater.

[CR55] Zhou Y, Liu X, Wang J (2019). Characterization of microplastics and the association of heavy metals with microplastics in suburban soil of central China. Sci Total Environ.

[CR56] Zhou B, Wang J, Zhang H (2020). Microplastics in agricultural soils on the coastal plain of Hangzhou Bay, east China: multiple sources other than plastic mulching film. J Hazard Mater.

[CR57] Zhou J, Gui H, Banfield CC (2021). The microplastisphere: biodegradable microplastics addition alters soil microbial community structure and function. Soil Biol Biochem.

